# The Timing of Semantic Processing in the Parafovea: Evidence from a Rapid Parallel Visual Presentation Study

**DOI:** 10.3390/brainsci12111535

**Published:** 2022-11-12

**Authors:** Silvia Primativo, Danila Rusich, Marialuisa Martelli, Lisa S. Arduino

**Affiliations:** 1 Department of Human Science, LUMSA University, 00193 Rome, Italy; 2 Department of Psychology, La Sapienza University, 00185 Rome, Italy

**Keywords:** parafoveal processing, semantic, word frequency, rapid parallel visual presentation, reading models

## Abstract

In the present investigation we adopted the Rapid Parallel Visual Presentation Paradigm with the aim of studying the timing of parafoveal semantic processing. The paradigm consisted in the simultaneous presentation of couple of words, one in fovea (W1) and one in parafovea (W2). In three experiments, we manipulated word frequency, semantic relatedness between the two words and the effect of stimulus duration (150, 100, 50 ms). Accuracy on W2 was higher when W1 and W2 were both of high-frequency and when they were semantically related. W1 reading times were faster when both words were highly-frequent but only when the two words were semantically related (150 ms); when W2 was highly frequent and semantically related to the foveal word (100 ms). When the stimuli were presented for 50 ms, the reading times were reduced when W1 was highly frequent and, crucially, in case of a semantic relation between the two words. Our results suggest that it is possible to extract semantic information from the parafovea very fast (within 100 ms) and in parallel to the processing of the foveal word, especially when the cognitive load required for the latter is reduced, as is the case for high-frequency words. We discuss the resulting data in terms of word recognition and eye movements’ models.

## 1. Introduction

Reading is an everyday task and a very complex activity at the same time. While reading a text, the eyes move from left to right in order to fixate content words and comprehend the sentence. The necessity to move the eyes is that fixated words are projected in the foveal region (subtending a visual angle of 2°), which is known to be characterized by highest visual acuity, generally comprising 6–8 characters. This region is generally distinguished from the parafoveal region, which extends 2–5° of the visual angle beyond the foveal region for up to approximately 15–20 characters [1]. We know that readers can also extract useful information from the parafovea, despite a decrease in visual acuity, lower attentional resources and reduced visual span [2,3]. A central issue in language processing research concerns the nature and amount of information that can be extracted from the parafovea during any given fixation and how and when the parafoveal information is integrated with the foveal information. A large set of empirical evidence has consistently shown an early processing of the parafoveal orthographic and phonological information. On the contrary, the evidence for semantic parafoveal processing is contradictory. The issue is crucial in light of the long-standing debate in terms of parallel vs. serial models of reading aloud. Indeed, the results reporting a lack of semantic processing of the parafoveal word [4,5] have been taken as evidence of seriality in word reading and eye movements models [6]. The literature reporting semantic parafoveal-on-foveal effects (e.g., [7,8,9]) report that words in the parafovea might be fully processed and their meanings immediately integrated and that this is suggestive of a possible parallel processing of two adjacent words. 

In this work, we focus on the results of our previous study where we provided evidence of parafoveal semantic processing, in order to better understand the timing of the mechanism underlying such processing. 

In [9], the Rapid Parallel Visual Presentation paradigm was adopted in order to investigate parafoveal semantic processing. In this study, two words were simultaneously presented for 150 ms, one in fovea and one in parafovea. The written word frequency (high and low) and the semantic relation between the two words were manipulated, while other visual and lexical variables were matched. The results showed a parafoveal-on-foveal semantic effect: the presence of a semantic relation between the two words reduced the reading times of the foveal word and increased accuracy on the parafoveal word. This paradigm has a strong potential in allowing a strictly controlled manipulation of the perceptual and lexical features of the stimuli. The results of the study did not allow to make inferences about the timing of the extraction of the semantic information from the non-fixated parafoveal word. Indeed, it could be the case that the semantic level was reached in our study because the duration of the stimuli presentation (150 ms) was long-enough to complete the processing of both the foveal and the parafoveal words, or it might have happened before. Focused on the strong benefits of the RPVP paradigm and aiming at investigating the timing of the lexical and semantic processing, in the present study, we manipulated the stimuli presentation duration. We presented the stimuli for 150 ms (experiment 1), 100 ms (experiment 2) and 50 ms (experiment 3). We also orthogonally manipulated the lexicality of the stimuli and the semantic relatedness between the foveal and the parafoveal words. 

By following the hypothesis of possible high-level processing in parafoveal view, only few studies have been devoted to investigate the timing of parafoveal semantic processing. In 2005, Inhoff et al. [10] evaluated the onset of linguistic information extraction in the parafovea by using the parafoveal display change paradigm in a sentence reading task. Each pre-target word was semantically related to the target one (e.g., MoRnInG-cOfFeE). Six viewing conditions were created: the control condition (where both words were continuously visible during sentence reading) and another five experimental conditions where the target location (parafovea) was occupied by a pseudoword at the onset of sentence reading. Then, the uninformative pseudoword was replaced with the target parafoveal word at different timing conditions after the onset of the participant’s fixation on the pre-target word. The results from this experiment revealed identical target viewing durations in the control condition and at the 70 ms delay. In the other conditions, the different preview delays corresponded to an increase of target viewing durations. Accordingly, the authors placed the acquisition of information from the parafoveal target preview from 70 to 140 ms after the onset of the fixation on the pre-target word. However, while this result suggests an early extraction of linguistic information from the parafoveal word, it does not clearly indicate whether the delay in the presentation of the parafoveal target word is linked with an impeded lexical rather than semantic processing. Indeed, the study did not involve semantic manipulation of the foveal and parafoveal words. As such, it is not possible to draw conclusions regarding the nature of the information extracted from the parafovea. 

Other studies (e.g., [11,12]) reported evidence of time-dependent facilitation due to semantic relatedness through the use of the parafoveal fast priming technique [13]. In this paradigm, the parafoveal target position is initially occupied by a random-letter string, which is replaced by a prime when the participant’s gaze crosses an invisible boundary located directly prior to the space preceding the pre-target word. Then, during the pre-target fixation, the target replaces the prime. Hohenstein et al. [11] manipulated the time of the prime duration (i.e., 35, 80, or 125 ms) and the semantic relation between prime and target (semantically related or unrelated). Results showed a semantic preview benefit for prime durations of 125 ms: first fixation and gaze duration on the target word were reduced when the prime and the target were semantically related. However, it has to be noted that all the pre-target words were highly-frequent and this could have facilitated the semantic pre-processing. 

Pan et al. [12], by using the same paradigm, manipulated the prime duration and its semantic relatedness with the parafoveal target during the oral reading of Chinese sentences. The authors obtained shorter fixation duration on the target words when the prime was semantically related to the target and was briefly presented (80 ms; Experiments 1 and 3). The results suggest that high-level semantic information can be obtained from parafoveal words within the 80 ms time-framework, at least in the Chinese language. 

In the present study, we will directly assess the time course of linguistic information extraction from a parafoveal word prior to its fixation by manipulating at the same time lexical and semantic variables.

The paradigm will allow us to measure (1) the parafoveal preview benefit, in terms of the accuracy advantage in reading a parafoveal word when it is semantically related vs. unrelated to the foveal word and (2) the parafoveal-on-foveal effect, in terms of faster reading times of the foveal word when this is semantically related vs. unrelated to the parafoveal word. This approach will enable the evaluation of both the independent and the combined effect of lexical and semantic effects, which is critical for the implementation of any model of word recognition and eye movements. 

## 2. Materials and Methods

### 2.1. Stimuli

One hundred twenty-eight pairs of Italian words, selected from the LEXVAR database [14], were used as stimuli. The written word frequency (mean = 133.4, range = 1–2253, s.d. = 252.9) and semantic relationship between the two words were manipulated orthogonally. The stimuli had a mean length of 6.21 letters (range 4–9). Words were of low (1–20 occurrences per million words) and of high (>51 occurrences per million words) adult written word frequencies, using a frequency count of 1,500,000 words drawn from written Italian texts (Istituto di Linguistica Computazionale, 1989). Two lists were created, one made up of semantically related (SR) and one semantically unrelated (SU) words, each consisting of 64 pairs of words. The two lists of words were matched by length in letters, age of acquisition, familiarity, imaginability, concreteness, adult written frequency, number of orthographic neighbors, bigram frequency and number of syllables (two tailed *t*-tests, all *p* > 0.05). Each list was composed of pairs of words labelled W1 for the word presented in the fovea and W2 for the word presented in the parafovea. Half of the stimuli were high frequency words and half were low frequency words. The semantic relation between words was previously established through the administration of a self-report questionnaire (7-point Lickert scale) to 62 participants (age range = 18–26 years, mean age = 19.4, s.d. = 1.3, F:M = 49:13) who did not take part in the main experiments. Participants were asked to rate on a scale from 1 to 7 the semantic relatedness of the two words. Semantically related words were categorized as being significantly more related (mean = 5.4, s.d. = 0.8, Min–Max = 3.1–6.7) compared to semantically unrelated words (mean = 1.5, s.d. = 0.4, Min–Max = 1.0–2.9), t2 (126) = 34.8, *p* < 0.001. Synonyms and antonyms were excluded from the set of stimuli pairs.

### 2.2. Software and Apparatus

The Experiment Builder software (SR Research Ltd., Mississauga, ON, Canada) was used for programming and running the experiment. In order to ensure the fixation stability and retinal position of the stimuli prior to their presentation on the screen, eye movements were recorded via an SR Research Ltd. Eye Link 1000 eye-tracker, sampling at 1000 Hz. Participants were seated at a 60 cm distance from the display, with their heads held firm by the use of a chin-rest. Stimuli were presented on a 17-inch LED screen (1366 × 768 pixel, 60 Hz). Participants’ voices were recorded via a one-way microphone connected to an external sound card (M-track 22).

### 2.3. Experimental Procedure

A nine-point calibration and validation procedures were performed at the beginning of each experiment. A drift correction was also run before each individual trial in order to ensure fixation stability. Subsequently, a fixation cross (subtending a visual angle of 0.5) was presented on the left-hand side of the screen. After a fixation on the fixation cross lasting for at least 250 ms, this disappeared and the stimuli (W1 and W2) appeared on the screen. The fixation cross was positioned between the second and the third letters of the foveal word (the optimal viewing position, [15]). The order of presentation of the pair of words was randomized across participants. 

The words were presented one in the fovea region (W1) and one in the parafovea region (W2). W1 and W2 were simultaneously shown on the screen for 150 ms (Experiment 1), 100 ms (Experiment 2) and 50 ms (Experiment 3). The words were presented in the Courier New font, a font with constant center-to-center letter spacing independent of the letter width. Each letter subtended a visual angle of 0.5°. The spatial extension of the stimuli, extending from 1° left of the fixation cross to the right side, ranged from a visual angle of 4.5° (both words short) to 9.5° (both words long). After the stimuli presentation, a blank screen appeared until the participant’s verbal response (no mask was used). The participants’ task was to read both words aloud. Speed and accuracy were both stressed in the instructions. Reaction times were measured for W1 (W1 reading onset) and accuracy was measured for W1 and W2. 

### 2.4. Participants

Different participants took part in the three experiments described below. Participants were undergraduate students and all gave written informed consent for their participation in the study. All participants reported themselves to be non-dyslexic and had normal or corrected-to-normal sight. The participants were naive with regard to the final purpose of the experiment. Thirty individuals took part in Experiment 1 (average age: 24 years; range = 19–31 years, s.d. = 4.2, F:M = 20:10). Twenty-five individuals were enrolled in Experiment 2 (mean age: 24 years old; range = 20–30 years, s.d. = 2.5, F:M = 15:10) and twenty took part in Experiment 3 (mean age: 24 years; range = 20–30 years, s.d. = 2.9, F:M = 10:10).

### 2.5. Data Cleaning and Statistical Procedure

Accuracy and reaction times were taken into account as dependent measures. We excluded from the analysis the participants that, because of low accuracy or recording failures, did not have data values in at least one of the experimental conditions. We also excluded trials containing saccades directed towards the parafoveal word. Saccades were detected by visual inspection of each individual trial. Accuracy on W2 and RTs on W1 were analyzed only for trials where W1 was accurately named. RTs higher or lower than 2.5 standard deviations from the subject’s mean RTs were excluded from the analysis. Accuracy and RTs were analyzed by repeated measures analyses of variance (ANOVAs), with three within-subjects factors (W1 written frequency, W2 written frequency and semantic relation between the two words). Significant interactions were further explored by using LSD post-hoc tests. Effect sizes are reported (partial eta squared, ηp2).

## 3. Results

### 3.1. Experiment 1—150 Msec

Three participants and 3.2% of the remaining observations were excluded because of the exclusion criteria described above. 

#### 3.1.1. Accuracy

**Figure 1:**
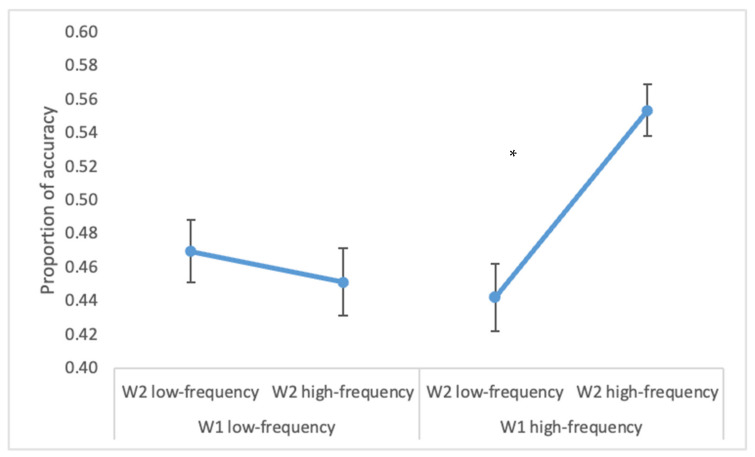
Experiment 1. W2 accuracy in the conditions of W1 and W2 low- and high-frequency. The figure represents the statistically significant interaction W1 frequency × W2 frequency. Bars represent standard errors. The asterisk (*) indicates statistically significant differences.

The overall accuracy on W1 was 94% (range = 88–100%) and on W2 it was 58% (range = 27–92%). A higher accuracy for W2 was observed for high- vs. low-frequency W1 (50 vs. 46%; F(1, 26) = 14.3, *p* < 0.00001, ηp2 = 0.35), for high- vs. low-frequency W2 (50 vs. 45%; F(1, 26) = 17.9, *p* < 0.0005, ηp2 = 0.41) and when there was a semantic relation between the two words (54% vs. 42%; F(1, 26) = 64.5, *p* < 0.001, ηp2 = 0.71). Moreover, the statistically significant interaction W1 × W2 frequency (F(1, 26) = 25.5, *p* < 0.00005, ηp2 = 0.50) indicated that when W1 was low-frequent, the frequency of the parafoveal word did not impact on accuracy (*p* = 0.3), while when W1 was high-frequent, the high-frequency of W2 increased accuracy in W2 (*p* < 0.001, see Figure 1). 

#### 3.1.2. Reaction Times

**Figure 2:**
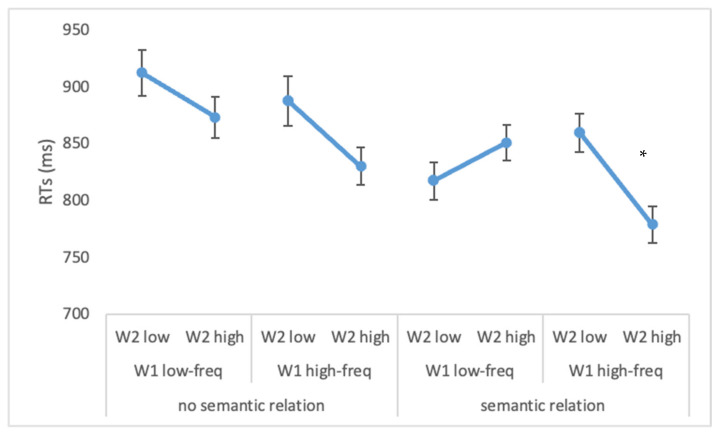
Experiment 1. Mean vocal reaction times (ms) for the semantic related and unrelated conditions as a function of W1 and W2 frequencies. The figure represents the interaction between W1 frequency × W2 frequency × semantic relation. Bars represent standard errors. The asterisk (*) indicates statistically significant differences.

Results showed shorter RTs for high- vs. low-frequent W1 (839 vs. 863 ms F(1, 26) = 7.1, *p* < 0.05; ηp2 = 0.21), when W2 was of a high frequency rather than a low frequency (833 vs. 869 ms F(1, 26) = 14.1, *p* < 0.00001; ηp2 = 0.35) and when there was a semantic relation between the two words (827 vs. 876 ms; F(1, 26) = 14.9, *p* < 0.00001; ηp2 = 0.37). Furthermore, the significant interaction between W1 frequency × W2 frequency × semantic relation reported in Figure 2 (F(1, 26) = 7.8, *p* < 0.0001; ηp2 = 0.23) showed faster reading times when the two words were highly-frequent, but only in the semantic relation condition (*p* < 0.01). 

#### 3.1.3. Discussion

The results from the analysis of accuracy in Experiment 1 revealed that participants were more accurate in reading the parafoveal word when the foveal and the parafoveal words were high-frequency words and when there was a semantic relation between the two stimuli. Thus, our results suggest lexical-semantic parafoveal pre-processing during the 150 msec of visual stimuli presentation. The reaction times revealed the presence of parafoveal-on-foveal effect both at lexical and semantic levels. Indeed, readers were faster in reading the stimuli if these were both highly frequent, but only when the two were semantically related. 

### 3.2. Experiment 2—100 Msec

In order to investigate the timing related to the emergence of the semantic processing in parafovea, the same experimental design and stimuli of experiment 1 was maintained, but stimulus presentation was reduced to 100 ms. In experiment 2, two participants and 2.2% of observations were excluded from data analysis because of the exclusion criteria described above. 

#### 3.2.1. Accuracy

**Figure 3:**
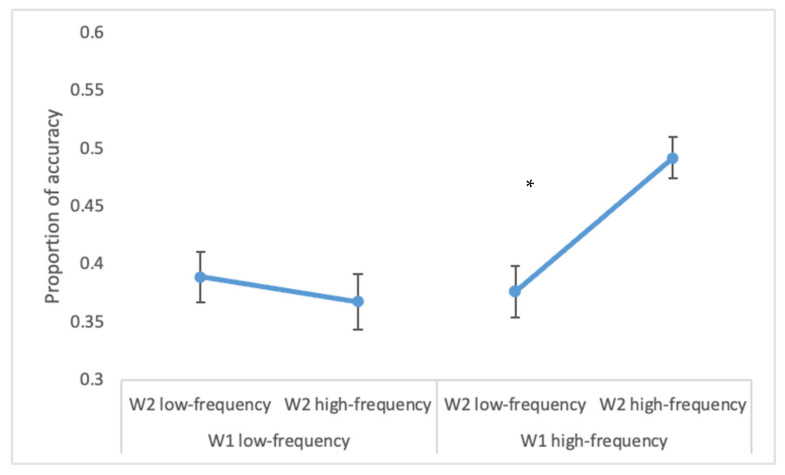
Experiment 2. Accuracy on W2 is reported in terms of the statistically significant interaction W1 frequency × W2 frequency. Bars represent standard errors. The asterisk (*) indicates statistically significant differences.

The overall accuracy on W1 was 98% (range = 94–100%) and on W2 it was 51% (range = 27–92%). A higher accuracy for W2 was observed when W1 was a high- rather than low-frequency word (43% vs. 38%; F(1, 22) = 23.11, *p* < 0.0001, ηp2 = 0.51), when W2 was a high- rather than low-frequency word (42% vs. 38%; F(1, 22) = 17.99, *p* < 0.0005, ηp2 = 0.45) and when there was a semantic relation between the two words (46% vs. 35%; F(1, 22) = 93.85, *p* < 0.0001, ηp2 = 0.81). Moreover, the interaction of W1 × W2 frequency was statistically significant (F(1, 22) = 25.54, *p* < 0.0001, ηp2 = 0.54; see Figure 3). The interaction indicated that when W1 was low-frequent, the frequency of the parafoveal word did not impact on accuracy (*p* = 0.28), while when W1 was high-frequent, the high-frequency of W2 increased W2 accuracy (*p* < 0.001). 

#### 3.2.2. Reaction Times

**Figure 4:**
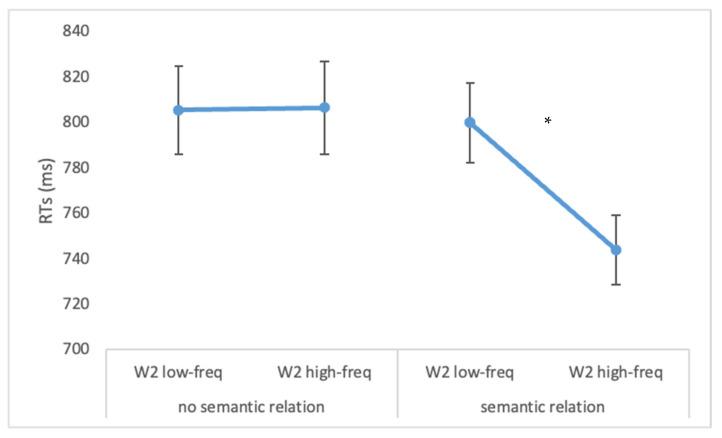
Reaction times for Experiment 2. The statistically significant interaction of semantic relation condition × W2 frequency is reported. Bars represent standard errors. The asterisk (*) indicates statistically significant differences.

Results showed shorter RTs for high vs. low-frequency W1 (776 vs. 801 ms F(1, 22) = 6.42, *p* < 0.05, ηp2 = 0.23), for high vs. low-frequency W2 (775 vs. 803 ms; F(1, 22) = 14.95, *p* < 0.0001, ηp2 = 0.40) and when there was a semantic relation between the two words (772 vs. 806 ms; F(1, 22) = 14.98, *p* < 0.0001, ηp2 = 0.41). The significant interaction W1 frequency × W2 frequency (F(1, 22) = 4.29, *p* < 0.05, ηp2 = 0.16) strengthened the result on accuracy: only when W1 was high-frequent, shorter reaction times was observed with high-frequent vs. low frequent W2 (*p* = 0.004). Crucially, the significant interaction W2 frequency × semantic relation (F(1, 22) = 13.44, *p* < 0.005, ηp2 = 0.38) emerged. As shown in Figure 4, the interaction indicated that the frequency of W2 influenced RTs, but only in the condition of a semantic relation between the two words. In particular, when W2 was a high frequency word, shorter reaction times were observed on W1 as compared to when W2 was a low-frequent word (744 vs. 800 ms, *p* < 0.0001). 

#### 3.2.3. Discussion

In experiment 2, the stimuli were presented for 100 ms. The results indicated that accuracy for parafoveal words was higher when the parafoveal word was semantically related to the foveal words and when both were highly frequent. RTs on the foveal word indicated a semantic parafoveal-on-foveal effect, with faster reading times for high-frequency parafoveal words only in the presence of a semantic relation between the two words. 

### 3.3. Experiment 3—50 Msec

Results from experiments 1 and 2 consistently indicated both lexical and semantic processing of parafoveal words when these were presented for 150 and 100 ms. In order to further investigate the timing of the semantic parafoveal on foveal effect, we repeated the experiment on a different sample of participants by reducing the duration of the stimulus presentation to 50 ms. Five participants and 2.2% of observations were excluded from data analysis because of the exclusion criteria described above. 

#### 3.3.1. Accuracy

**Figure 5:**
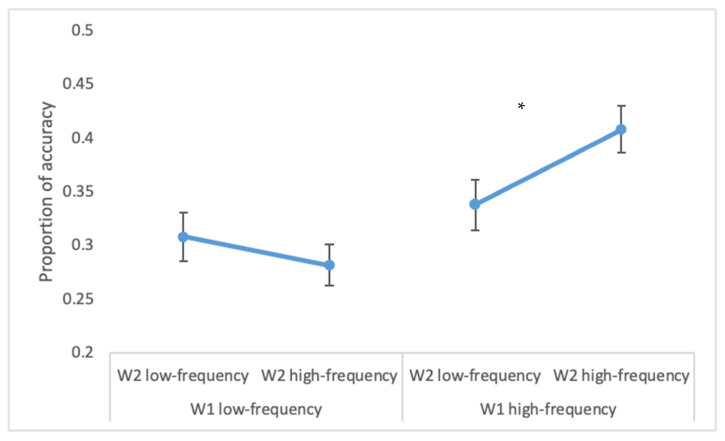
Experiment 3. Accuracy on W2 is reported in terms of the statistically significant interaction W1 frequency × W2 frequency. Bars represent standard errors. The asterisk (*) indicates statistically significant differences.

Overall accuracy on W1 was 95% (range = 90–100%) and overall accuracy on W2 was 39% (range = 19–66%). A higher accuracy for W2 was observed when W1 was of high vs. low frequency (37% vs. 29%; F(1, 14) = 23.85, *p* < 0.0005, ηp2 = 0.63) and when there was a semantic relation between the two words (38% vs. 28%; F(1, 14) = 62.38, *p* = 0.0000, ηp2 = 0.82). Moreover, the two-way interactions of W1 × W2 frequency was statistically significant (F(1, 14) = 9.13, *p* < 0.0001, ηp2 = 0.39, see Figure 5). The interaction indicated that when W1 was low-frequent, the frequency of the parafoveal word did not impact on accuracy (*p* = 0.18), while when W1 was high-frequent, the high-frequency of W2 increased accuracy in W2 (*p* = 0.002). 

#### 3.3.2. Reaction Times

Results showed shorter RTs when W1 was of high frequency (773 vs. 821 ms F(1, 14) = 6.69, *p* < 0.05, ηp2 = 0.32) and when there was a semantic relation between the two words (778 vs. 816 ms; F(1, 14) = 6.31, *p* < 0.05, ηp2 = 0.31). The main effect of W2 frequency did not reach statistical significance despite the data being consistent with expectations of faster reading times for high vs low-frequency words (730 vs. 761 ms, *p* = 0.1). No statistically significant interactions emerged.

#### 3.3.3. Discussion

Results from experiment 3 indicated higher accuracy for the parafoveal word when this was semantically related to the foveal word and when both were highly frequent. Results also indicated that reading times on the foveal words were influenced by the frequency of the foveal word and, crucially, by the semantic relation with the parafoveal word. 

**Figure 6:**
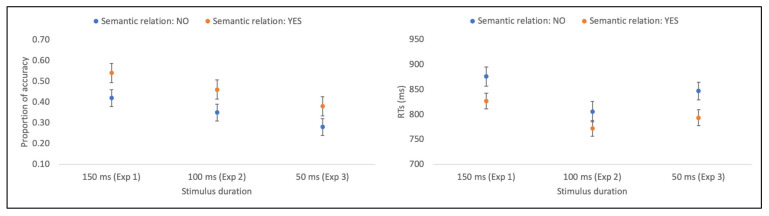
Summary of results in terms of accuracy and response times in case of presence or absence of semantic relation between W1 and W2. Bars represent standard errors.

A summary of the results from the three experiments is reported in Figure 6, for both accuracy and response times, showing a more efficient processing of the parafoveal word when it is semantically related to the foveal word. This is the case for all the three tested experimental conditions and thus when the stimulus duration is 150, 100 and 50 ms. 

## 4. Discussion

In the present study, we investigated the time course of both lexical and semantic processing in parafovea during word-pairs reading in Italian. To this aim, we adopted the RPVP paradigm, by using two semantically related or unrelated words presented for 150 ms, 100 ms and 50 ms (experiments 1, 2 and 3, respectively). The two words were simultaneously presented on the screen, one in the fovea and the other in the parafovea, and participants were asked to read both of them aloud. 

The analysis of accuracy on the parafoveal word in relation to the lexical and semantic manipulations is indicative of the parafoveal preview benefit. Results were coherent across all the three experiments: participants were more accurate in reading the parafoveal word when the foveal and the parafoveal words were both of high-frequency and when there was a semantic relation between the two stimuli. 

Results of W1′s reaction times represent a crucial metric of the parafoveal-on-foveal effect. When the stimuli were presented for 150 ms (experiment 1), we observed faster reading times when both words were highly-frequent, but only in the semantic relation condition. When the stimuli were presented for 100 ms (experiment 2), faster reaction times were observed when the parafoveal word (W2) was highly frequent and semantically related to the foveal word. Finally, when the stimuli were presented for 50 ms (experiment 3), the reading times were reduced when the foveal word (W1) was highly frequent and, crucially, in the condition of semantic relation between the two stimuli. 

Our results shed some new light on the timing of the multiple high-level processing of the parafoveal word during reading, despite they cannot be generalized to natural reading. In particular, it is interesting to note that, differently from lexicality, a semantic processing of the parafoveal word was already present at the shortest duration (50 ms). This was not the case for longer stimulus durations (100 and 150 ms) where, instead, reaction times were modulated by both lexicality and semantic status of W2. This result was unexpected and deserves some comments. First, it could be an artefact of time constraining and of the list compositions, where 50% of the pair of stimuli were semantically related. Secondly, it could be possible that participants have adopted a guessing strategy which resulted in being mainly successful in the semantic relation condition compared to the lexical one. This issue should be further addressed in future studies. 

Nonetheless, results clearly suggest an extremely fast processing of lexical and semantic components (within 100 ms), indicating that these two components are integrated (or extracted in parallel) within a very short amount of time. This result is in accordance with previous data in different languages such as German and Chinese. In fact, Ref. [11] reported a semantic preview benefit within 80 ms for German reading. Additionally by experimentally controlling the parafoveal preview exposure duration, Ref. [12] showed that semantic information can be extracted from the parafoveal word in only 80 ms in Chinese. On the other hand, the semantic preview benefit has been more elusive in English. It might be speculated that the parafoveal processing up to high-levels (i.e., semantic) is influenced by intrinsic orthographic peculiarities, such as the level of transparency of a language. In fact, it has been suggested that the word semantic access might be reached later in orthographies with opaque grapheme–phoneme conversion rules such as English (as compared to more transparent ones like German or Italian). In this case, the parafoveal preview is too brief in order to provide enough information to allow semantic access [16]. 

Such evidence of parallel word processing and parafoveal linguistic influences during the early stages of reading is in line with processing gradient models such as the SWIFT [17]. In this model, the attentional gradient covers stimuli in both foveal and parafoveal regions of the visual field, which are in turn processed in parallel. The attentional gradient may decrease when foveal processing is difficult and requires additional resources, and increase when the foveal word is easy to process and a surplus of foveal resources remains. In this latter case, the attentional gradient should cover a larger region of the visual field (Foveal load hypothesis; [18]). Accordingly, the present results may indicate that some attentional resources are assigned to the upcoming word. When the foveal word is of high rather than low frequency, the parafoveal word is processed faster and to a higher-cognitive degree. This supports the claim that the attentional gradient adapts to the foveal load because a fixated low frequency word should draw more resources, narrowing the attentional spotlight and diminishing parafoveal processing [19]. Moreover, the semantic relation between the two words facilitates word recognition. Hence, parafoveal information extraction does not depend on completion of foveal word processing, but occurs in parallel to foveal information extraction (and processing). The SWIFT model is well accounted in the recent OB1-Reader model [20], where the parallel word recognition features achieve the simultaneous processing of perceptual, lexical and semantic information from parafoveal vision. On the contrary, sequential models as the E-Z Reader [21] can hardly explain the fast and simultaneous achievement of high-level information from the parafovea as reported in our study. The E-Z Reader model, indeed, proposed many subsequent steps of word processing, such as (1) a pre-attentive level, where the first low-level word information (as length) should be processed; (2) the first level of lexical access (L1), where the frequency and predictability of a word impact word processing speed and finally (3) the second level of lexical access where the meaning of a word can be achieved (L2). In this model, the saccade programming is determined by the attention allocation. Hence, after L2, the attention is shifted to word n + 1 in 50 ms from the end of the last lexical access level. This mechanism may explain the parafoveal processing (N + 1) when eyes still fixate word N. Accordingly, the recent hybrid mechanism of the saccade triggering model [22] incorporates the serial assumption of the E-Z Reader model [21]. In this model, the readers plan the saccadic movements before complete processing of the word, and the parafoveal information can ease processing during reading via trans-saccadic integration (short fixations on a target word when the preview is more similar to it) or forced fixations (short fixations on words that would be skipped independent of the target). Our data can be smoothly explained within such a hybrid model. Indeed, the relatively low accuracy rate of the parafoveal word seems to suggest that while the semantic processing is initiated in parafoveal vision, it is not necessarily fully accomplished. 

We acknowledge that our study has some limitations. In particular, the RPVP procedure does not resemble natural reading and thus conclusions cannot be further generalized over the adopted experimental procedure. Despite both speed and accuracy being stressed in the instructions to participants, response times were longer as compared to single word reading. This might be strictly linked to the paradigm itself: indeed, differently from single word reading paradigms, in our case, it is explicitly required to process the two words that are shown simultaneously and to verbally report both of them. It is thus possible that the coding process and the planning of a verbal response is nearly doubled, hence increasing response times. 

## 5. Conclusions

In conclusion, we showed that it is possible to extract semantic information from the parafovea in parallel to the processing of the foveal word, especially when the cognitive load required for the latter is reduced, as is the case for high-frequency words. Furthermore, the process of acquiring lexical and semantic information from the parafovea is extremely fast, within 100 ms. We believe that these results are noteworthy and should be taken into account in the evaluation and development of models of word recognition and eye movements. 

## Data Availability

The data supporting the findings of this study are available upon reasonable request.
